# Ablation targets of scar-related ventricular tachycardia identified by dynamic functional substrate mapping

**DOI:** 10.1186/s43044-023-00414-w

**Published:** 2023-10-13

**Authors:** Mohammad Gamal Elewa, Sherif Altoukhy, Haitham Abdelfattah Badran, Hayam El Damanhoury, John Kamel Zarif

**Affiliations:** Cardiology Department, Ain Shams University Hospital, Faculty of Medicine, Ain Shams University, 5B – Swiss Project B, PO 11826, Nasr City, Cairo Egypt

**Keywords:** Catheter ablation, Substrate mapping, Decrement-evoked potential, Cardiomyopathy, Ventricular tachycardia

## Abstract

**Background:**

Dynamic functional substrate mapping of scar-related ventricular tachycardia offers better identification of ablation targets with limited ablation lesions. Several functional substrate mapping approaches have been proposed, including decrement-evoked potential (DEEP) mapping. The aim of our study was to compare the short- and long-term efficacy of a DEEP-guided versus a fixed-substrate-guided strategy for the ablation of scar-related ventricular tachycardia (VT).

**Results:**

Forty consecutive patients presenting for ablation of scar-related VT were randomized to either DEEP-guided or substrate-guided ablation. Late potentials were tagged and ablated in the non-DEEP group, while those in the DEEP group were subjected to RV extrastimulation after a drive train. Only potentials showing significant delay were ablated. Patients were followed for a median duration of 12 months. Twenty patients were allocated to the DEEP group, while the other 20 were allocated to the non-DEEP group. Twelve patients (60%) in the DEEP group had ischemic cardiomyopathy versus 10 patients (50%) in the non-DEEP group (*P*-value 0.525). Intraoperatively, the median percentage of points with LPs was 19% in the DEEP group and 20.6% in the non-DEEP group. The procedural time was longer in the DEEP group, approaching but missing statistical significance (*P*-value 0.059). VT non-inducibility was successfully accomplished in 16 patients (80%) in the DEEP group versus 17 patients (85%) in the non-DEEP group (*P* value 0.597). After a median follow-up duration of 12 months, the VT recurrence rate was 65% in both groups (*P* value 0.311), with a dropout rate of 10% in the DEEP group. As for the secondary endpoints, all-cause mortality rates were 20% and 25% in the DEEP and non-DEEP groups, respectively (*P*-value 0.342).

**Conclusions:**

DEEP-assisted ablation of scar-related ventricular tachycardia is a feasible strategy with comparable short- and long-term outcomes to a fixed-substrate-based strategy with more specific ablation targets, albeit relatively longer but non-significant procedural times and higher procedural deaths. The imbalance between the study groups in terms of epicardial versus endocardial mapping, although non-significant, warrants the prudent interpretation of our results. Further large-scale randomized trials are recommended. Trial registration: clinicaltrials.gov, registration number: NCT05086510, registered on 28th September 2021, record https://classic.clinicaltrials.gov/ct2/show/NCT05086510

## Background

Over the last few decades, catheter ablation of scar-related ventricular tachycardia (VT) has witnessed remarkable evolution and has gained much credit as a cornerstone in VT management [1].

The vast majority of scar-related ventricular tachycardias pose a significant hemodynamic burden on patients presenting for catheter ablation of VT. This limits the role of activation and entrainment mapping for the identification of ablation targets, given the necessity of induction of sustained VT [2]. Moreover, the critical isthmus of VT is often difficult to identify; a single patient may have multiple scars, and a single scar may house multiple reentrant circuits. Substrate mapping has emerged as a feasible strategy to overcome such challenges by identifying and targeting low-voltage areas as well as regions with split, fractionated, or late local potentials during intrinsic rhythm [3–5].

Notably, substrate-based strategies entail extensive ablation of abnormal potentials, although a substantial number of these may not be incorporated in reentry circuits [6]. Further, it has been demonstrated that abnormal potentials may be buried within the QRS complex in sinus rhythm and manifest only with premature stimulation when tissue conduction properties are at stake [7]. This led to the hypothesis that dynamic functional substrate mapping during ventricular extrastimulation might permit better identification of ablation targets with a limited number of ablation lesions.

Several functional substrate mapping approaches have been proposed, including decrement-evoked potential (DEEP) mapping among others [8–13]. Despite proving feasibility in the clinical setting, none of these strategies have been compared to fixed-substrate mapping strategies in terms of short- and long-term outcomes.

The aim of our study was to compare the efficacy of a DEEP-guided ablation strategy versus that of a fixed-substrate ablation strategy for the ablation of scar-related VT in terms of acute success rates as well as long-term VT recurrence rates.

## Methods

### Patients

In this randomized single-blinded prospective clinical trial, consecutive patients presenting to XXX university hospitals for ablation of ventricular tachycardia were evaluated for participation in the study. The patient recruitment period was from October 2021 till January 2023. Approval was obtained from the ethical committee at XXX university before starting the research.

- *Inclusion criteria*
  - Patients with structural heart disease and sustained monomorphic VT documented by 12-lead electrocardiogram (ECG) or implantable cardioverter defibrillator (ICD) electrograms resistant to antiarrhythmic drug treatment or requiring ICD therapies.
- *Exclusion criteria*
  - Patients with ventricular arrhythmias that were attributed to reversible causes.

### Methods

On admission, patients were subjected to the following after a written informed consent:

#### Randomization

Patients were randomized in a single-blinded fashion.

#### Procedure

The electrophysiological study and ablation were conducted under general anesthesia. Continuous non-invasive or intra-arterial blood pressure monitoring and digital pulse oximetry were performed. ICD therapies were inactivated.

All mapping and ablation procedures were performed by the same operator who is an expert in VT ablation with an average volume of 20 procedures per year. A steerable quadripolar or decapolar catheter was positioned in the right ventricular apex or coronary sinus. The access was via endocardial retroaortic approach, trans-septal approach, epicardial approach, or endocardial retroaortic with ad hoc epicardial approach. Epicardial mapping and ablation were performed when preprocedural ce-CMR showed epicardial scar; endocardial mapping failed to identify subendocardial scars, unipolar LV mapping suggested the presence of epicardial scar, ECG of clinical or induced VT suggested epicardial origin, or as a first-line approach in patients with non-ischemic dilated cardiomyopathy [14, 15]. The right ventricle (RV) was mapped when a right ventricular origin of VT was suspected, e.g., in arrhythmogenic right ventricular cardiomyopathy (ARVC). Surface electrocardiograms (ECGs) and bipolar intracardiac electrograms were continuously monitored.

#### Mapping and ablation strategy

All patients underwent bipolar voltage mapping during sinus rhythm. Mapping was performed by either an irrigated tip ablation catheter (ThermoCool SF™ catheter; Biosense Webster, Diamond Bar, CA, USA), (ThermoCool ST™ catheter; Biosense Webster, Diamond Bar, CA, USA), (FlexAbility ™; Abbott, St. Paul, MN, USA), (TactiCath™; Abbott, St. Paul, MN, USA), or a multi-electrode mapping catheter (Pentaray™, Biosense Webster, Diamond Bar, CA, USA). Late potentials (LPs) (defined as sharp, high frequency, or fractionated potentials at or after the terminal portion of QRS [16]) were pinpointed and tagged.

**Fig. 1:**
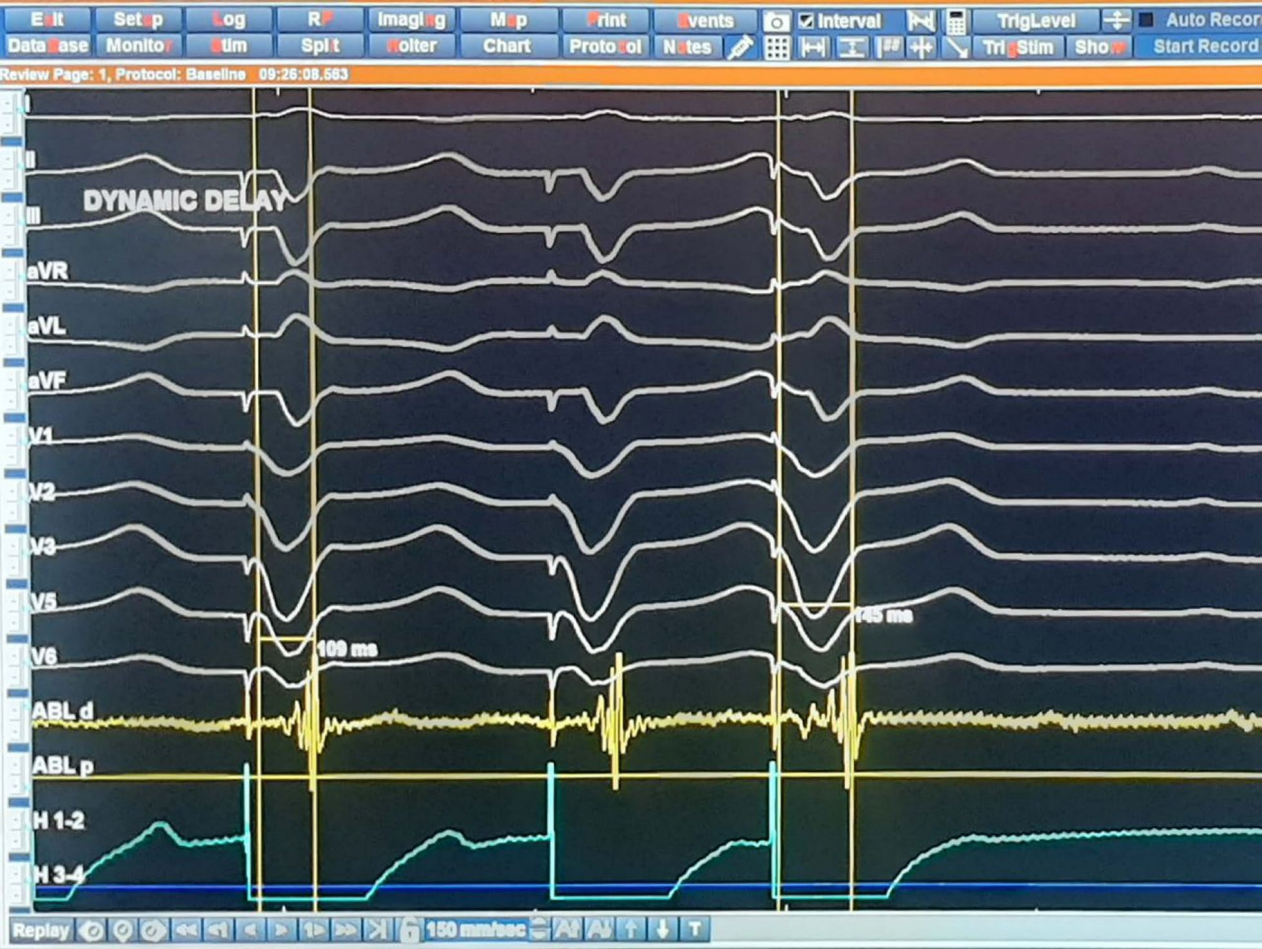
Intracardiac electrogram demonstrating DEEP. Intracardiac electrogram of a patient with arrhythmogenic right ventricular cardiomyopathy during right ventricular S1–S2 pacing. The late component of the paced QRS on the distal pole of the ablation catheter shows significant delay with extrastimulation denoting a DEEP

Patients allocated to the DEEP group were subjected to further analysis of their LPs by RV extrastimulus pacing. This constituted an S1 drive train at 600 ms or 500 ms (according to the sinus rhythm cycle length) followed by a single S2 extrastimulus at 20 ms longer than the ventricular effective refractory period. Thereafter, the time interval from the surface ventricular far-field signal to the local bipolar LP electrogram was measured both during the RV drive train and with S2 extrastimulation. If the difference between the 2 measured values was > 10 ms, the analyzed LP was annotated as a DEEP. The same applied to multicomponent signals where DEEP was annotated if there was a more than 10-ms splitting of components in response to S2 (Fig. 1) [8]. The percentage of points with LPs and DEEPs was calculated in relation to the total number of mapped points.

Concerning the DEEP group, ablation was initially restricted to points with DEEPs, while in the non-DEEP group, ablation aimed at the elimination of all LPs. Acute success was defined as VT non-inducibility, including both clinical and non-clinical tachycardias. VT was still inducible after ablation, activation, and entrainment mapping were performed for hemodynamically tolerated tachycardias, aiming at the identification of the critical components of the re-entrant circuit according to previously defined criteria [17, 18], followed by ablation at such locations. If the VT was not tolerated, pace mapping was performed aiming at a > 96% match [19]**.**

#### Follow-up

The study population was followed up for a median duration of 12 months for the occurrence of the study endpoints. The primary endpoint was VT recurrence rates. Secondary endpoints were all-cause mortality and cardiovascular mortality. For patients with implanted defibrillators, follow-up was done by interrogation of device recordings, while when defibrillators were not implanted, follow-up was done by history taking for the occurrence of symptoms or need for emergency department admission attributed to recurrence of VT.

#### Statistical analysis

Continuous variables with normal distribution are presented as mean ± standard deviation and range, while those with non-normal distribution are reported as median and interquartile range (IQR). Continuous variables were compared by Student’s t test, while categorical variables were compared by Chi-square tests or Fisher’s exact tests as appropriate. Statistical analysis was performed using SPSS 25 for Windows (SPSS Inc, Chicago, Illinois, USA). A *P*-value < 0.05 was considered statistically significant.

## Results

This open-label randomized controlled trial included 40 patients presenting with scar-related VT for ablation. Twenty patients were allocated to the DEEP group, while the other 20 were allocated to the non-DEEP group.

### Baseline characteristics (Table 1)

**Table 1:** Comparison between the DEEP group and the non-DEEP group regarding baseline characteristics and pre-operative data

	DEEP group	Non-DEEP group	Test value	*P*-value
	No. = 20	No. = 20		
Age				
Mean ± SD	55.65 ± 15.87	53.75 ± 11.85	0.42	0.67
Range	18–76	30–71		
Sex				
Male	17 (85.0%)	19 (95.0%)	1.11	0.29
Female	3 (15.0%)	1 (5.0%)		
Smoking				
No	13 (65.0%)	12 (60.0%)	0.10	0.74
Yes	7 (35.0%)	8 (40.0%)		
Diabetes				
No	14 (70.0%)	16 (80.0%)	0.53	0.46
Yes	6 (30.0%)	4 (20.0%)		
Hypertension				
No	13 (65.0%)	14 (70.0%)	0.11	0.73
Yes	7 (35.0%)	6 (30.0%)		
Serum creatinine				
Mean ± SD	1.25 ± 0.61	1.18 ± 0.35	0.39	0.69
Range	0.7–3.5	0.6–2		
Etiology				
ICM	12 (60.0%)	10 (50.0%)	4.84	0.30
DCM	5 (25.0%)	10 (50.0%)		
CHD	1 (5.0%)	0 (0.0%)		
ARVC	1 (5.0%)	0 (0.0%)		
Non-compaction	1 (5.0%)	0 (0.0%)		
ICM				
Non-ICM	8 (40.0%)	10 (50.0%)	0.40	0.52
ICM	12 (60.0%)	10 (50.0%)		
Cardiac surgery				
No	19 (95.0%)	18 (90.0%)	0.36	0.54
Yes	1 (5.0%)	2 (10.0%)		
EF (%)				
Mean ± SD	34.55 ± 10.29	30.70 ± 7.95	1.32	0.19
Range	20–56	20–47		
Presentation				
Storm	13 (65.0%)	15 (75.0%)	4.64	0.09
Recurrent shocks	3 (15.0%)	5 (25.0%)		
Sustained VT	4 (20.0%)	0 (0.0%)		
Device				
No	5 (25.0%)	2 (10.0%)	2.10	0.34
ICD	9 (45.0%)	13 (65.0%)		
CRTD	6 (30.0%)	5 (25.0%)		
Pre-AADs				
Amiodarone only	0 (0.0%)	0 (0.0%)	3.33	0.34
BBs only	2 (10.0%)	0 (0.0%)		
Mexiletine only	0 (0.0%)	0 (0.0%)		
Amio + BBs	17 (85.0%)	17 (85.0%)		
BBs + Mexiletine	0 (0.0%)	1 (5.0%)		
Amio + BBs + Mexiletine	1 (5.0%)	2 (10.0%)		
Pre-operative shocks				
Mean ± SD	2.10 ± 1.25	2.50 ± 0.89	−1.16	0.25
Range	0–4	1–5		
Pre-operative therapies				
Mean ± SD	23.46 ± 10.27	22.89 ± 8.78	0.16	0.86
Range	11–41	12–36		

*AADs* anti-arrhythmic drugs, *ARVC* arrhythmogenic right ventricular cardiomyopathy, *BBs* beta-blockers, *CHD* congenital heart disease, *CRTD* cardiac resynchronization therapy defibrillator, *DCM* dilated cardiomyopathy, *DEEP* decrement-evoked potential, *EF* ejection fraction, *ICD* implantable cardioverter defibrillator, *ICM* ischemic cardiomyopathy, *SD* standard deviation, *VT* ventricular tachycardia

There were no significant differences in the demographics of the 2 groups.

There was no significant difference between the 2 groups regarding smoking, diabetes, hypertension, baseline serum creatinine, and history of cardiac surgery (Table 1). Concerning the etiology of VT, 12 patients (60%) in the DEEP group had ischemic cardiomyopathy as compared to 10 patients (50%) in the non-DEEP group (*P*-value 0.525). Non-ischemic patients in the non-DEEP group included 5 patients with dilated cardiomyopathy, 1 patient with ARVC, 1 patient with a history of congenital heart disease (post-Fallot repair), and 1 patient with biventricular non-compaction, while non-ischemic patients in the non-DEEP group all had dilated cardiomyopathy.

### Pre-operative data

Concerning pre-operative data, the 2 groups were similar as well. The mean ejection fraction (EF) was 34.55 ± 10.29% in the DEEP group and 30.70 ± 7.95% in the non-DEEP group. Thirteen patients (65%) in the DEEP group presented with VT storm as compared to 15 patients (75%) in the other group. A total of 8 patients (3 in the DEEP group and 5 in the non-DEEP group) presented with recurrent shocks. Only 4 patients presented with a single episode of sustained VT; all were allocated to the DEEP group. As for defibrillator implants, 75% of patients in the DEEP group (*n* = 15) had implanted defibrillators as compared to 90% (*n* = 18) in the non-DEEP group. Pre-operative anti-arrhythmic drugs were comparable as well. The mean number of pre-operative shocks was 2.10 ± 1.25 in the DEEP group and 2.50 ± 0.89 in the non-DEEP group (*P*-value 0.251). The burden of pre-operative defibrillator therapies was 23.46 ± 10.27 in the DEEP group versus 22.89 ± 8.78 in the non-DEEP group (*P*-value 0.869). Table 1 summarizes the pre-operative data in both groups.

### Intraoperative data: (Table 2)

**Table 2:** Comparison between the DEEP group and the non-DEEP group regarding intraoperative data

Intra-operative	DEEP group		Non-DEEP group		Test value	*P*-value
	No	%	No	%		
Access						
Endocardial	16	80.0	10	50.0	4.30	0.11
Epicardial–endocardial	4	20.0	9	45.0		
Epicardial	0	0.0	1	5.0		
Scar location						
Anterior + Septal + Apical	11	55.0	9	45.0	4.20	0.52
Inferior + Posterior + Lateral	2	10.0	6	30.0		
RV non-outflow	0	0.0	0	0.0		
Perimitral, outflow	2	10.0	2	10.0		
Aborted	1	5.0	0	0.0		
Anterior + Septal + Apical + Inferior + Posterior + Lateral	3	15.0	3	15.0		
RV non-outflow + Perimitral, outflow	1	5.0	0	0.0		

*DEEP* decrement-evoked potential, *RV* right ventricle

With respect to intraoperative data, Pentaray multi-electrode catheter was the mapping catheter in 6 patients, 3 in each group. The access to LV or RV was endocardial in 16 patients (80%) in the DEEP group and 10 patients (50%) in the non-DEEP group. Combined epicardial–endocardial access was elected in 4 patients (20%) and 9 patients (45%) in the DEEP and non-DEEP groups, respectively. Epicardial access alone was the access in only one patient in the non-DEEP group. Scar location, summarized in Table 2 together with other intraoperative data, was matched in the 2 study groups (*P*-value 0.52).

### Mapping and ablation data: (Fig. 2)

The number of mapped points was comparable between the 2 groups. The median percentage of points with LPs was 19% in the DEEP group (IQR 12.8–31.7) and 20.6% in the non-DEEP group (IQR 10.1–32.05) (*P*-value 1). In the DEEP group, there was a significant difference between the number of points with LPs vs DEEP (19 vs. 5.8%). This was reflected as a highly significant statistical difference between the median number of ablation points between the DEEP and non-DEEP groups (Median = 30, IQR 22–44) versus (Median = 81, IQR 55.5–129.5), respectively (Table 3). Procedural time was longer in the DEEP group vs the non-DEEP group (261 vs. 196.4 min), a figure that just missed statistical significance (*P*-value 0.059). The mean fluoroscopy time was 30.1 min for the DEEP group and 23.1 min in the non-DEEP group (*P*-value 0.13). The mean ablation time was 12.3 min for the DEEP group and 32.8 min in the non-DEEP group (*P*-value < 0.001).

**Fig. 2:**
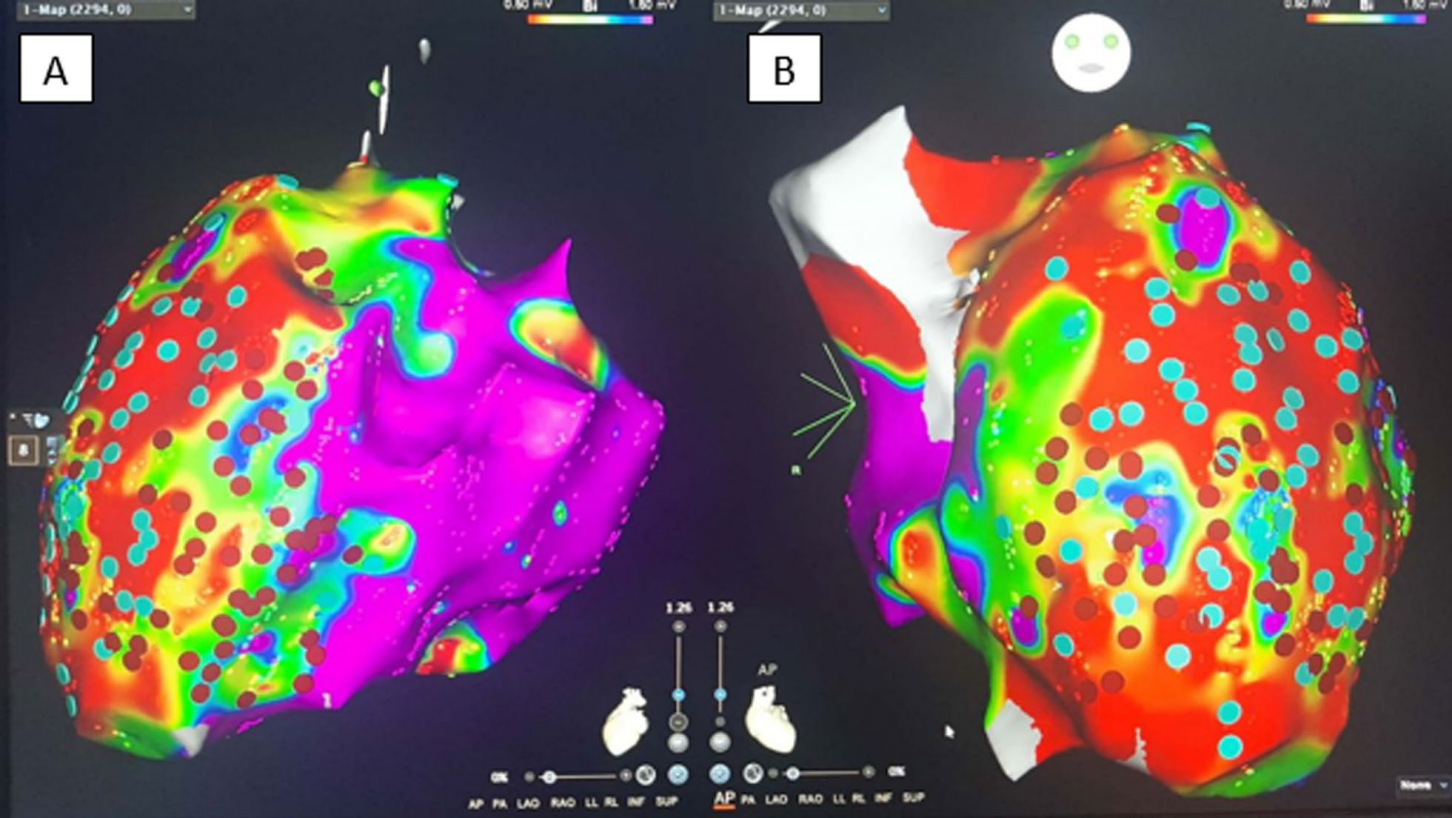
A bipolar voltage electroanatomical endocardial substrate map in left lateral (**A**) and antero-posterior (**B**) views showing DEEPs and LPs. Purple color indicates normal voltage (>1.5 mV), red color indicates low voltage (<0.5 mV), and the colors in between indicate borderline voltage between 0.5 mV and 1.5 mV. Navy blue tags refer to late potentials with no delay (non-DEEPs), while red tags refer to DEEPs that were subsequently ablated

**Table 3:** Comparison between the DEEP group and the non-DEEP group regarding mapping and ablation data

Intra-operative	DEEP group	Non-DEEP group	Test value	*P*-value
	No. = 20	No. = 20		
Procedure time (minutes)				
Mean ± SD	261.00 ± 117.78	196.40 ± 89.87	1.95	0.05
Range	60–430	90–398		
Mapped points				
Median (IQR)	362 (308.5–1211.5)	399 (218.5–653.5)	-0.78	0.43
Range	36–3722	97–2300		
LP (%)				
Median (IQR)	19 (12.8–31.7)	20.6 (10.1–32.05)	0.00	1.00
Range	4.8–50.3	3.5–60.6		
DEEP (%)				
Median (IQR)	5.8 (1.9–7.3)	–	–	–
Range	1–21.6	–		
No. of ablation points				
Median (IQR)	30 (22–44)	81 (55.5–129.5)	-3.54	< 0.001
Range	3–111	11–200		

*DEEP* decrement-evoked potential, *IQR* interquartile range, *LP* late potential, *SD* standard deviation

### Procedural endpoints

VT non-inducibility was successfully accomplished in 16 patients (80%) in the DEEP group versus 17 patients (85%) in the non-DEEP group (*P* value 0.597). Three patients in each group still had the clinical VT inducible at the end of the procedure, and the procedure was aborted due to the occurrence of complications, while only 1 patient in the DEEP group had an inducible VT other than the clinical one. Concerning procedural complications, 85% of patients in each group were complication-free at the end of the procedure. Two patients died in the DEEP group, while there were no deaths in the non-DEEP group. In the DEEP group, 2 patients had cardiac tamponade, and 1 had cardiac tamponade together with iatrogenic AV block. Among the 3 patients with cardiac tamponade, 1 occurred during initial trans-septal puncture due to left atrial appendage perforation, and the procedure was aborted before mapping, another patient had a perforated LV apical aneurysm, and the third had an aortic cusp perforation. All 3 patients underwent urgent pericardiocentesis and urgent surgical intervention; the first one survived, while the other 2 did not. Regarding complications in the non-DEEP group, 1 patient had acute pulmonary edema and was urgently resuscitated, another had ventricular fibrillation (VF) and received an external shock, and a third had iatrogenic atrioventricular (AV) block. Fortunately, all patients in the non-DEEP group survived. The differences in complication and procedural mortality rates between the 2 groups were not statistically significant (*P* values 0.306 and 0.147, respectively). Table 4 summarizes procedural endpoints.

**Table 4:** Comparison between the DEEP group and the non-DEEP group regarding procedure endpoint, complication, procedural mortality

Postoperative	DEEP group		Non-DEEP group		Test value	*P*-value
	No	%	No	%		
Procedure endpoint						
All VTs non-inducible	16	80.0	17	85.0	1.03	0.59
Clinical VT non-inducible, others inducible	1	5.0	0	0.0		
Clinical VT inducible	3	15.0	3	15.0		
Complication						
None	17	85.0	17	85.0	6.00	0.30
Tamponade	2	10.0	0	0.0		
Acute pulmonary edema	0	0.0	1	5.0		
VF	0	0.0	1	5.0		
AV block	0	0.0	1	5.0		
Tamponade + AV block	1	5.0	0	0.0		
Procedural mortality						
No	18	90.0	20	100.0	2.10	0.14
Yes	2	10.0	0	0.0		

*AV* atrio-ventricular, *VT* ventricular tachycardia, *VF* ventricular fibrillation

### Primary and secondary endpoints (Table 5)

**Table 5:** Comparison between the DEEP group and the non-DEEP group regarding study endpoints

	DEEP group	Non-DEEP group	Test value	*P*-value
	No. = 20	No. = 20		
Recurrence				
Non-recurrence	5 (25.0%)	7 (35.0%)	2.33	0.31
Recurrence	13 (65.0%)	13 (65.0%)		
Lost follow-up	2 (10.0%)	0 (0.0%)		
All-cause mortality				
No	14 (70.0%)	15 (75.0%)	2.14	0.34
Yes	4 (20.0%)	5 (25.0%)		
Lost follow-up	2 (10.0%)	0 (0.0%)		
Cardiovascular mortality				
No	14 (70.0%)	15 (75.0%)	2.14	0.34
Yes	4 (20.0%)	5 (25.0%)		
Lost follow-up	2 (10.0%)	0 (0.0%)		
Time to 1st VT (ms)				
Median (IQR)	3 (0.5–9)	3 (2–5)	-0.07	0.93
Range	0–13	0.5–11		
Follow-up duration (ms)				
Median (IQR)	12 (5–13)	12 (8.5–12)	-0.43	0.66
Range	0–26	3–24		

*DEEP* decrement-evoked potential, *IQR* interquartile range, *VT* ventricular tachycardia

After a median follow-up duration of 12 months, the VT recurrence rate was 65% in both groups, with a dropout rate of 10% in the DEEP group (*P* value 0.311). As for the secondary endpoints, all-cause mortality rates were 20% and 25% in the DEEP and non-DEEP groups, respectively (*P*-value 0.342). The cause of death was VT recurrence in 3 patients (60%) in the DEEP group and 4 patients (80%) in the non-DEEP group. One patient in each group died due to advanced heart failure and cardiogenic shock. The mean EF at follow-up was 33.5% in the DEEP group and 30.15% in the non-DEEP group with no significant change from baseline in either group (*P*-value 0.73 for DEEP group and 0.81 for non-DEEP group).

## Discussion

The principal findings of our study are:

1. DEEP-assisted ablation of scar-related ventricular tachycardia is a feasible strategy with comparable acute success rates to fixed-substrate-based techniques.
2. Although VT recurrence rates in our study after an average follow-up period of 12 months were as high as 65%, the rates are equal in the DEEP-guided and LP-guided groups, denoting the absence of significant difference between the 2 strategies in terms of long-term efficacy.
3. DEEP-guided ablation of scar-related VT is associated with rates of all-cause mortality and cardiovascular mortality as well as time to first VT, which are similar to a fixed substrate-guided strategy. However, the procedural mortality was higher in the DEEP group (10% vs. 0%), a difference which, despite being non-significant, warrants further evaluation in large RCTs. Longer procedural times in this arm might have been a contributor.
4. In addition, there is a significant reduction in the number of ablation points, which is at the expense of a longer procedural time. The difference in mean procedural time between the DEEP and the non-DEEP groups was 65 min, which is a considerable figure despite missing statistical significance. Further experience with the technique, in addition to utilizing novel mapping features are expected to remarkably shorter procedural time.

To our knowledge, this is the first randomized double-armed prospective trial addressing the feasibility of DEEP-guided ablation of scar-related ventricular tachycardia in a head-to-head comparison with a fixed-substrate-centered strategy. Traditionally, conventional mapping techniques, namely activation and entrainment mapping, have been the gold standard for VT ablation [20]. With 70% of VTs being non-inducible or hemodynamically not tolerated, several substrate-based techniques have been devised as an alternative strategy, targeting EGM abnormalities during stable rhythm (sinus rhythm or paced rhythm).

Jaïs et al. demonstrated the feasibility and safety of targeting local abnormal ventricular activity (LAVA) elimination as a procedural endpoint with a significant reduction in the combined endpoint of VT recurrence or death [21]. Elimination of abnormal potentials has been compared to conventional mapping techniques and has proven superiority in terms of long-term recurrence (15.5% vs. 48.3%, respectively; log-rank *p* < 0.001) [22]. A recurrence rate of 41.4% was reported in another trial after a mean follow-up duration of 3.14 years postablation, but with a significant reduction in the burden of ICD shocks and VTs even in the patient cohort that experienced VT recurrence [23]. Another single-armed prospective trial of LP abolition strategy has shown an acute success rate of 71.4% and a long-term VT recurrence rate of 20% [24]. Of note, the recurrence rate reached 75% in patients with incomplete LP abolition. Incomplete LP abolition is an independent predictor of VT recurrence and is attributed to a septal location of the substrate with proximity to the conductive system, higher LV mass, and the use of conventional—as compared to high-density—mapping catheters [25]. In our study, the LP elimination arm had an acute success rate of 85%, which is midway between that reported by Vergara et al. (71.4%) [24] and Roca-Luque et al. (71%) on one side [25] and that reported by Luigi et al. (100%). [22].

Although effective, substrate mapping protocols are not without limitations. The identification of abnormal EGMs is prone to inter-observer variability. Besides, targeting all abnormal electrograms can lead to extensive ablation that often involves myocardial areas not incriminated in the VT circuit. The deleterious effect of such a strategy on the global ventricular function has not been ruled out, although in our study there was no significant reduction in the mean EF after 12 months in either group. Over and above, unnecessary ablation lesions can theoretically create areas of partial myocardial viability and slow conduction, calling forth new substrates for reentry. Functional substrate mapping techniques have been introduced with the aim of achieving optimal short- and long-term outcomes with the least number of ablation lesions.

In this paper, we compared a DEEP-guided functional mapping strategy to conventional static substrate mapping. The theory behind DEEP mapping is based on the observation that decrement precedes unidirectional functional block leading to reentry, as has been demonstrated in atrial tissue specimens [26]. The mechanism was reproduced in infarcted human hearts where unidirectional block preceded the initiation of sustained monomorphic VT [27].

The electro-physiologic implication of this notion has been demonstrated in the mechanistic study by Jackson et al., where DEEPs were found to colocalize with the diastolic corridor of VT with higher specificity than late potentials with no significant difference in sensitivity [28]. Such findings paved the way for testing such a strategy in the clinical setting. Porta-Sanchez et al. performed DEEP-guided ablation of VT in 20 patients with ischemic cardiomyopathy [8]. The percentage of LPs and DEEPs were 16.8% and 4.8%, respectively, which are comparable to ours. Likewise, the rate of VT non-inducibility was 80%. At a 6-month follow-up, 75% of patients were free from VT compared to 60% in the DEEP arm of our study.

Although a DEEP-guided ablation strategy is plausible, it inherently has several downsides. First of all, repeated pacing in patients with a depressed ventricular function that is aggravated by recurrent VT episodes is not without risk. Furthermore, electrophysiologically speaking, the magnitude of delay is subject to several variables, including the pacing location, the characteristics of the conducting channel as well as the coupling interval of the extrastimulus [29, 30]. Stimulation in the vicinity of the scar tissue from the side opposite to the entrance site produces longer delays. Shorter delays occur with pacing at farther sites from the scar and sites close to conduction barriers [29]. Potentials in unprotected conducting channels with multiple side branching have shorter DEEP delays compared to protected channels with fewer additional side branches [29].

A recently published study has shown that delivering an S2 at 400 ms and using a 20-ms decrement threshold for defining DEEP resulted in improved specificity for the identification of VT isthmuses in 13 patients with ischemic VT without compromising sensitivity, leading to a 59% reduction in the area targeted for ablation. At the 6-month-follow-up, freedom from device-detected VTs was 92% [30]. The paced electrogram feature analysis (PEFA) technique accounts for the electrogram duration as well as latency in response to RV apical stimulation at variable coupling intervals to identify VT isthmuses and has shown promising VT-freedom rates [13].

Another argument against the DEEP strategy is that drive train pacing before extra-stimulation allows for the adaptation of sodium channels [31], a phenomenon that may mask the abrupt changes in conduction properties that occur in the real world when single extrasystoles initiate VTs. In 2020, Srinivasan et al. introduced the Barts Sense Protocol, which entails high-density mapping during sinus rhythm and with single-sensed extrastimuli [12]. Interestingly, the Barts Sense Protocol could identify an area of ablation that is larger than that identified during intrinsic rhythm, with a highly specific correlation to the critical isthmus (96%). The authors attributed such contradiction with DEEP mapping results to the advantage of the absence of tissue adaptation to extrastimulation with single-sensed beats [12]. With employing such a protocol, the rate of VT non-inducibility was 96%, and 90% of patients were free from VT at 12 months of follow-up.

### Limitations

The mean age in our study was 54 years, which is younger than the usual mean age in prior scar-related VT studies. A possible explanation is that our cohort included patients with congenital heart diseases, ARVC, and dilated non-ischemic cardiomyopathies. Such patient categories are generally younger when they present with VTs than the ischemic population represented in most of the published literature.

Besides, 45% of our patients had a presentation of VT storm. This is a remarkably high percentage, given that most of the time, VT ablation is performed on an elective basis for recurrent VT. This is correct for regions where the availability of resources is not problematic. However, in our country, where the resources for VT ablation are limited, patients presenting with VT storm have the highest priority, while patients with 1^st^ or recurrent VT episodes are given every trial of medical management before referral for elective ablation.

Our study has shown a 1-year VT recurrence rate of 65% after DEEP-guided ablation. Several factors are proposed to have contributed to such high rates. In our current study, the multi-electrode Pentaray catheter was only used in 15% of patients in each group. The remaining cases underwent point-by-point mapping using conventional bipolar catheters. In the study by Porta-Sanchez et al., mapping was performed by multipolar catheters in 80% of cases [8]. This factor might have contributed to the high recurrence rates in our study, given the published data that suggest a correlation between the density of mapping and ICD-therapy-free survival [32]. The ability of the catheter to detect abnormal electrograms depends on the size of electrodes, spacing between electrodes and wavefront direction in relation to bipolar pairs [33, 34].

Another possible cause of higher recurrence rates is that, unlike the study by Porta-Sanchez et al., our study included patients with non-ischemic etiology of VT (45% of the study sample and 40% of the DEEP arm). Long-term outcomes of VT ablation in such a cohort are generally less favorable [35]. Moreover, our patient population had a pre-procedural burden of shocks and ICD therapies that is to some extent higher than that in the study by Porta-Sanchez et al. This might highlight the possibility that our patients had a more aggressive disease that was subsequently translated to poorer outcomes. We believe that the relatively high procedural complication rate in our study (15%) is at least in part explained by the instability of our patients’ condition prior to and during the procedure.

Another crucial limitation of our study is that although randomized, the trial arms showed an imbalance in terms of endocardial versus epicardial mapping. More patients in the non-DEEP group underwent epicardial mapping, while the number of mapped points was similar between the 2 groups, which carries the possibility that the non-DEEP group had less dense mapping. This issue should be considered while drawing conclusions from our study.

Last but not least, our trial was single-centered with limited sample size. We recommend further large-scale multi-center randomized trials that compare the several available functional substrate mapping techniques, integrating the ever-developing mapping technology with our growing understanding of VT electrophysiology to obtain the optimum patient outcomes.

## Conclusions

DEEP-assisted mapping and ablation of scar-related ventricular tachycardia is a feasible strategy with comparable short- and long-term outcomes to a fixed-substrate-based strategy with more specific ablation targets, albeit relatively longer but non-significant procedural times and higher procedural deaths. The imbalance between the study groups in terms of epicardial versus endocardial mapping, although non-significant, warrants the prudent interpretation of our results. Further large-scale randomized trials that integrate recent mapping technologies with novel functional substrate mapping protocols are recommended to improve ablation outcomes.

## Data Availability

The datasets used and/or analyzed during the current study are available from the corresponding author upon reasonable request.

## Abbreviations

AADs: Anti-arrhythmic drugs
APO: Acute pulmonary edema
ARVC: Arrhythmogenic right ventricular cardiomyopathy
AV: Atrioventricular
BB: Beta-blocker
Ce-CMR: Contrast-enhanced Cardiac Magnetic Resonance
CHD: Congenital heart disease
CRTD: Cardiac resynchronization therapy defibrillator
CRTP: Cardiac resynchronization therapy pacing
DCM: Dilated cardiomyopathy
DEEP: Decrement-evoked potential
DM: Diabetes mellitus
EAM: Electro-anatomical map
ECG: Electrocardiogram
EDP: Evoked delayed potential
EF: Ejection fraction
HSC: Hidden slow conduction
HTN: Hypertension
ICD: Implantable cardioverter defibrillator
ICM: Ischemic cardiomyopathy
ILAM: Isochronal late activation mapping
IQR: Interquartile range
LAVA: Local abnormal ventricular activity
LP: Late potential
LV: Left ventricle
MI: Myocardial infarction
PEFA: Paced electrogram feature analysis
RSWMA: Resting segmental wall motion abnormality
RV: Right ventricle
SD: Standard deviation
SP: Sense protocol
VF: Ventricular fibrillation
VT: Ventricular tachycardia
